# Classical *Mus musculus* Igκ Enhancers Support Transcription but not High Level Somatic Hypermutation from a V-Lambda Promoter in Chicken DT40 Cells

**DOI:** 10.1371/journal.pone.0018955

**Published:** 2011-04-20

**Authors:** Naga Rama Kothapalli, Darrell D. Norton, Sebastian D. Fugmann

**Affiliations:** Laboratory of Molecular Biology and Immunology, Molecular Immunology Unit, National Institute on Aging, National Institutes of Health, Baltimore, Maryland, United States of America; The Rockefeller University, United States of America

## Abstract

Somatic hypermutation (SHM) of immunoglobulin genes is initiated by activation-induced cytidine deaminase (AID) in activated B cells. This process is strictly dependent on transcription. Hence, *cis*-acting transcriptional control elements have been proposed to target SHM to immunoglobulin loci. The *Mus musculus* Igκ locus is regulated by the intronic enhancer (iE/MAR) and the 3′ enhancer (3′E), and multiple studies using transgenic and knock-out approaches in mice and cell lines have reported somewhat contradictory results about the function of these enhancers in AID-mediated sequence diversification. Here we show that the *M. musculus* iE/MAR and 3′E elements are active solely as transcriptional enhancer when placed in the context of the *IGL* locus in *Gallus gallus* DT40 cells, but they are very inefficient in targeting AID-mediated mutation events to this locus. This suggests that either key components of the *cis*-regulatory targeting elements reside outside the murine Igκ transcriptional enhancer sequences, or that the targeting of AID activity to Ig loci occurs by largely species-specific mechanisms.

## Introduction

Antibody diversity is hallmark of vertebrate immune function, and is generated during two distinct stages of B cell development. First, the assembly of immunoglobulin (Ig) gene segments by V(D)J recombination gives rise to functional antigen receptor genes and the primary antibody repertoire. Secondary diversification is achieved through the processes of somatic hypermutation (SHM), Ig gene conversion (GCV) and class switch recombination (CSR) to generate high-affinity antibodies with improved effector function. All three processes, SHM, GCV and CSR, are initiated by activation-induced cytidine deaminase (AID) [1], [2], [3]. This enzyme deaminates cytosine residues within single-stranded DNA that is transiently generated during transcription of Ig genes [4]. The resulting G:U mismatches are resolved by direct replication or by DNA repair mechanisms involving uracil DNA glycosylase, mismatch repair factors, and error-prone DNA polymerases [5]. GCV copies sequence information from upstream pseudogenes into the variable region of the Ig gene resulting in “templated” nucleotide changes, whereas SHM creates individual “non-templated” point mutations. SHM occurs across all jawed vertebrates, while GCV has been only observed in selected species, including *G. gallus* and *Oryctolagus cuniculus* [6]. Although the end products of SHM and GCV differ, it is widely believed that the molecular principles that govern their AID-dependent initiation phase are identical [7]. As AID is highly mutagenic, specific mechanisms are in place to restrict its activity to Ig gene loci [8]. Nonetheless, it is worth noting, that non-Ig genes can become targets of widely distributed low levels of SHM [9], as AID interacts with components of the general transcription machinery [10]. The focusing of SHM/GCV to Ig genes is thought to be mediated by *cis*-regulatory elements, and given the mechanistic link between transcription and SHM/GCV/CSR, transcriptional enhancers were considered to be strong candidates [11], [12].

We and others recently reported first evidence for the existence of such *cis*-regulatory targeting elements in the context of the endogenous *G. gallus IGL* gene locus, and based on their location it was conceivable that they reside within transcriptional enhancers [13], [14], [15]. The situation with respect to the identity and location of such targeting elements in the *M. musculus* Igκ gene is less clear. This locus contains three enhancers: the intronic enhancer (iE/MAR) [16], the 3′ enhancer (3′E) [17], and the downstream enhancer (Ed) [18]. Transgenic studies indicated that the iE/MAR and 3′E elements are necessary for SHM of Igκ transgenes [19]. In contrast, knock-out mice in which the iE/MAR or the 3′E were deleted individually showed normal or only modestly (2- to 2.5-fold) reduced levels of SHM, respectively [20]. The latter could be readily explained by a corresponding drop in Igκ transcription in these mice [20]. This suggested that neither of these enhancers individually is necessary for SHM, and hence that there might be functional redundancy. Simultaneous deletion of both enhancers resulted in a block of B cell development as Vκ-Jκ rearrangement was inhibited [21], and hence the SHM status could not be addressed. A recent report, however, showed that the presence of both *M. musculus* Igκ enhancers is sufficient to drive SHM of respective GFP reporter transgenes in DT40 cells [22]. In this case, the E-box motifs (CAGGTG) within these enhancers were critical for targeting, but did not play a role in transcription.

To resolve these controversial observations regarding the importance of the iE/MAR and 3′E for targeting of SHM to Ig loci, we performed cross-complementation experiments in which the endogenous *cis*-regulatory elements essential for transcription and targeting in the *IGL* locus of DT40 cells were replaced with the respective *M. musculus* enhancers. Here we report that the *M. musculus* enhancers are fully competent to drive transcription in the context of the *G. gallus IGL* locus, but are unable to support high-levels of SHM/GCV, neither individually nor in combination. Our findings suggest that transcriptional enhancer function, even that of Ig enhancers, does not correlate with targeting of AID activity. This finding raises the possibility that additional binding sites important for efficient targeting in the *M. musculus* Igκ locus reside outside the defined iE/MAR and 3′E transcription enhancers. An alternative and even more intriguing explanation could be species specific targeting mechanisms for AID activity.

## Results

**Figure 1:**
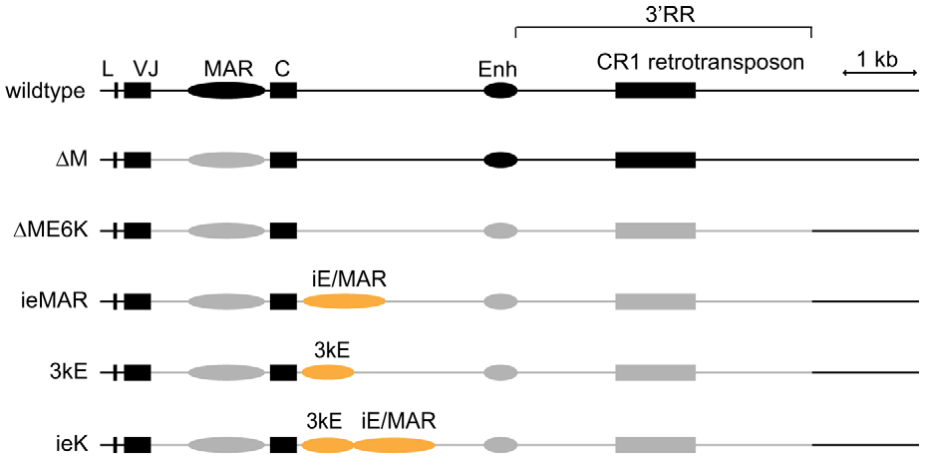
Schematic representation of the *IGL* loci in individual DT40 lines. The rearranged allele of the *IGL* locus in the indicated cell lines is drawn to scale. The leader (L), the VJ exon, constant region (C), and the CR1 retrotransposon (present in the published chicken genome as well as in DT40 cells, S.D.F. unpublished) are shown as filled boxes. The putative matrix attachment region (MAR) and the 467 bp enhancer (Enh) are shown as filled ovals. Elements that are present are shown in black, those that are deleted are in grey. The murine enhancer elements (iE/MAR and 3kE) in the respective knock-in alleles are depicted as orange ovals.

In our previous study, the ΔM cell line in which the VJ and C exons are joined showed robust SHM/GCV at levels comparable to that of wild-type cells [13]. The newly created VJC exon allowed us to generate gene-targeting constructs that target solely the “active” rearranged and not the “silent” unrearranged allele, and thus ΔM cells served as the parental line for subsequent deletion studies. In particular, we generated a DT40 cell line (ΔME6K) which completely lacks transcription and SHM/GCV in its *IGL* gene (Fig. 1) [13]. This phenotype was caused by a deletion of large portions of non-coding DNA from this locus including the 3′ regulatory region (3′RR) that harbors transcriptional enhancers and targeting elements for AID-mediated sequence diversification. Importantly, SHM/GCV was still occurring normally in the Ig heavy (*IGH*) chain locus of these cells, indicating that these cells were still competent for SHM/GCV. Thus, the ΔME6K background represents a good model system to test the functional properties of *M. musculus* Igκ enhancers in these AID-mediated processes in the context of an endogenous *IGL* locus.

**Figure 2:**
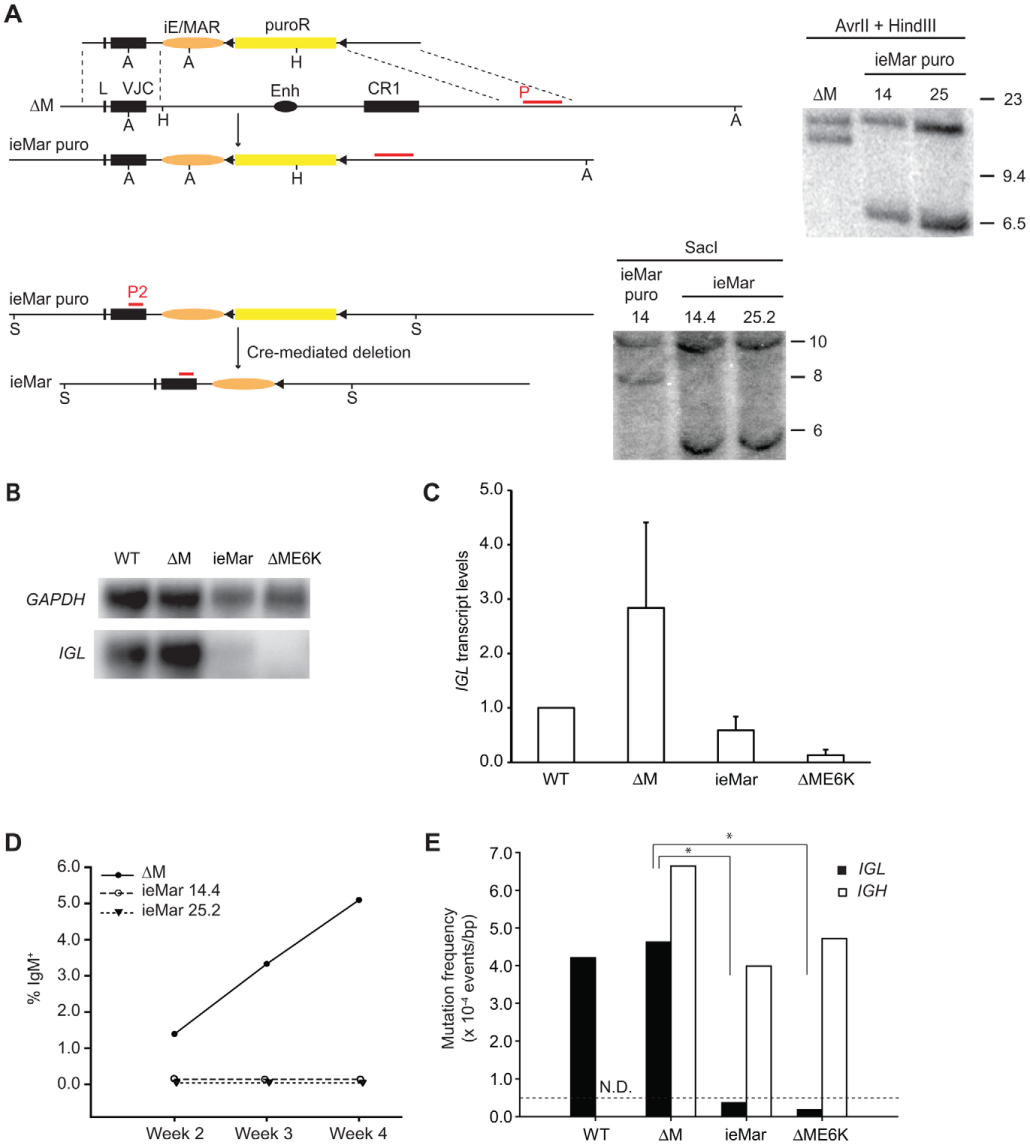
Functional analysis of the iE/MAR enhancer element. A) Schematic representation of the gene-targeting approach. The rearranged IGL allele of the parental ΔM line, the intermediate ieMAR puro allele, and the final product, the IGL locus of the ieMar DT40 cells, are drawn to scale. The targeting vector is shown with its homology arms, the iE/MAR (orange oval), the puromycin selection cassette (yellow box) and flanking loxP sites (filled triangles), but the plasmid backbone is omitted for clarity. The restriction sites used for the Southern blot analyses are marked (A: AvrII, H: HindIII, and S: SacI), and the probes for each step (P1 and P2, respectively) are shown as red lines above the locus. The asterisk in each Southern blot marks the band that originates from the non-rearranged allele. B) Northern Blots were run using 10 µg of total RNA of wild-type (WT) DT40 cells and each of the indicated cell lines, and hybridized sequentially with IGL and GAPDH probes. One representative example is shown. C) Steady-state transcript levels of IGL were measured by Northern blot and normalized to those of GAPDH. The level in wild-type (WT) cells was arbitrarily set to one, and each data point represents the average of at least four independent values per genotype. D) GCV was assessed by flow cytometry over four weeks starting from single cell clones. The average percentage of IgM+ cells in a total of 24 subclones from two independent ieMar lines is shown at each time point. E) Mutation event frequencies in the IGL (filled bars) and IGH (open bars) loci were determined by sequencing the VJ or VDJ exon from two independent subclones after four weeks of continuous culture. The background level of 0.5×10–5 events/bp for this system was determined in AID-deficient DT40 cells [23], and is shown as a dotted line. The data for wild type (WT) and ΔME6K were obtained from [13]. Asterisks indicate p<0.005 in a Student's t-test.

To determine the functional properties of the *M. musculus* iE/MAR with respect to transcription and SHM, iE/MAR knock-in DT40 cell lines were created in the context of the ΔME6K *IGL* genotype starting from ΔM cells (Fig. 1). The *IGL* locus in the ieMar DT40 cells matches that of ΔME6K, but in addition harbors the iE/MAR fragment from the *M. musculus* Igκ locus. Two independent clones with this genotype were generated by gene targeting, ieMar 14.4 and ieMar 25.2, and their identities were confirmed by Southern blot analysis (Fig. 2A). The puromycin-resistance selection cassette was subsequently removed using a cell-permeable Cre-recombinase. To test whether the *M. musculus* iE/MAR element provides transcriptional enhancer activity in the context of the *G. gallus IGL* locus, we measured the steady state *IGL* transcript levels by Northern blot analysis. While the ΔME6K line carries no endogenous *G. gallus IGL* enhancer and therefore shows no evidence of transcription [13], *IGL* transcripts were four-fold lower albeit readily detectable in the ieMar lines compared to the parental ΔM line (Fig. 2B and 2C). They are, however, still at the 59% of the levels found in wild-type cells (Fig. 1C), and importantly such reduced levels were found to be sufficient to support robust SHM/GCV (see clones ΔE and ΔME in [13]). This indicated that the *M. musculus* iE/MAR sequences are active as transcriptional enhancers in the *IGL* locus of DT40 cells, but clearly less strong than the cognate *G. gallus IGL* enhancer.

To test whether the iE/MAR enhancer is also sufficient to recruit AID-mediated sequence diversification in this system, twelve sub-clones of ieMar 14.4 and ieMar 25.2 were continuously grown for four weeks, and their surface IgM phenotype was assessed by flow cytometry at weeks two, three, and four. All cells started as IgM^−^ due to a premature stop codon in the VJ exon of their *IGL* gene that was introduced during gene-targeting. A subset of the GCV events leads to a reversion of this mutation, thereby creating surface IgM^+^ cells. In contrast to cultures of the parental ΔM line which showed increasing percentages of IgM^+^ cells over time, IgM^+^ cells remained undetectable in any of the 24 ieMar sub-clones even after 4 weeks (Fig. 2D). As some of the GCV and non-templated SHM events do not result in a reversion of the stop codon, we also determined the frequency of mutation events by sequencing the VJ exon of the *IGL* locus at the end of the four week culture. The mutation frequency of 0.363×10^−4^ events/bp is dramatically lower than that observed in the wild-type cells (4.07×10^−4^ events/bp, [13]), and is even below the background level of our system (0.5×10^−4^ events/bp, observed in AID-deficient DT40 cells, [23]) (Fig. 2E). This suggests that the iE/MAR is unable to support high levels of SHM/GCV in DT40 cells. One possibility is that these cells entirely lost their ability to diversify their Ig genes. Thus we sequenced the *IGH* locus of our ieMar cell lines at the four week time point as well. Importantly, the *IGH* gene showed robust evidence of AID-mediated sequence diversification (4.07×10^−4^ events/bp) similar to that observed in the parental ΔM cells (6.22×10^−4^ events/bp, [13]), suggesting that AID activity was unaltered in these knock-in clones (Fig. 2E). Based on the flow cytometry and the supporting DNA sequencing data, we conclude that the *M. musculus* iE/MAR enhancer is indeed unable to support efficient SHM/GCV of the DT40 *IGL* locus (i.e. detectable by our experimental system) despite its ability to act as a transcription enhancer. These findings are consistent with the phenotype of iE/MAR knock-out mice that showed largely unaltered SHM [20]. They are, however, in striking contrast to the initial observations made by Betz et al. using traditional Igκ transgenes [19].

**Figure 3:**
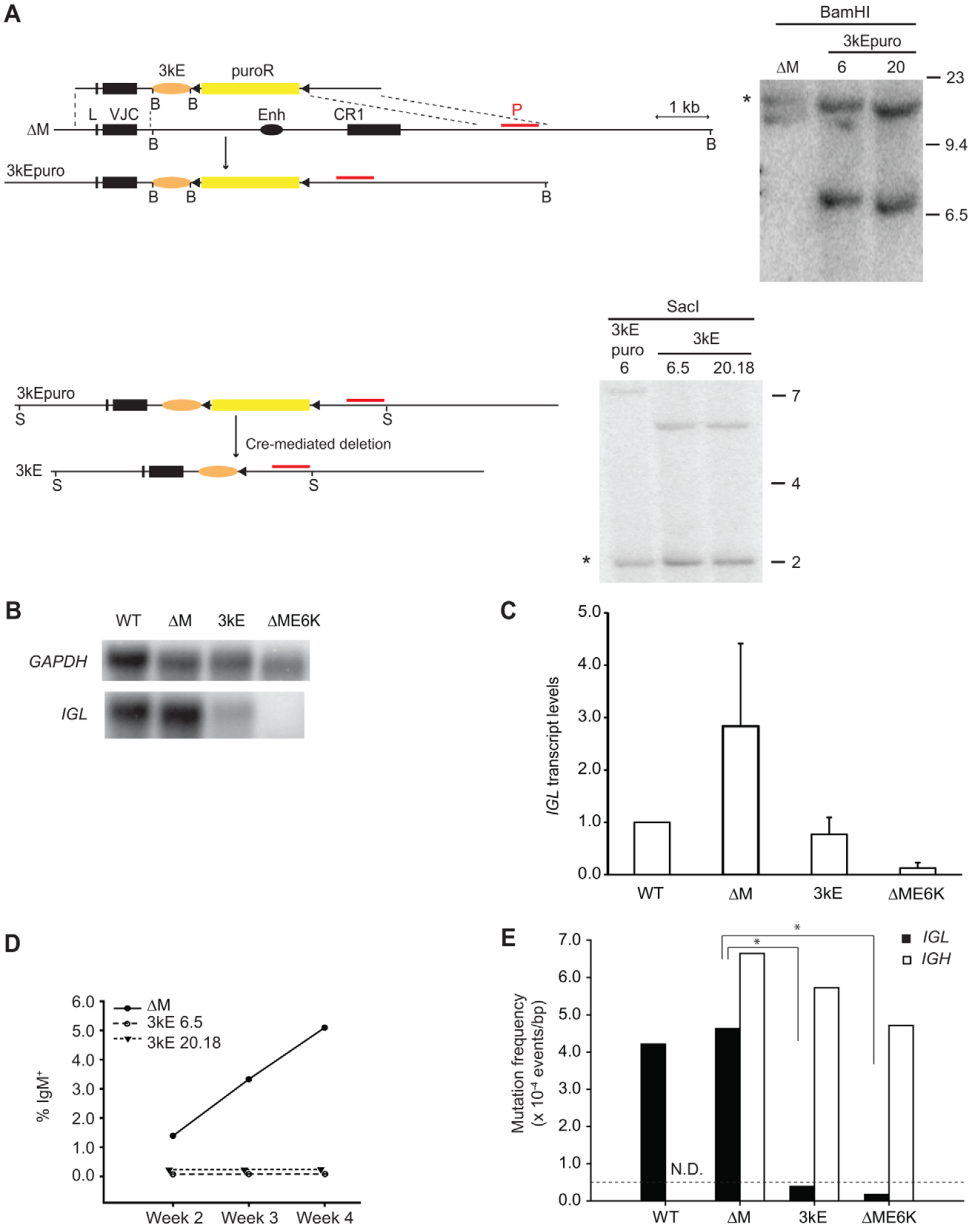
Functional analysis of the 3′E enhancer element. A) Gene-targeting scheme to generate the 3kE DT40 knock-in cell lines. The 3kE element is represented as an orange oval, the restriction sites used for Southern Blot analyses are marked (B: BamHI, and S: SacI), and the Southern blot probe (red line, P) used for genotyping is shown. For a description of all other elements, see legend to Fig. 1. The asterisk in each Southern blot marks the band that originates from the non-rearranged allele. B) Northern Blots were run using 10 µg of total RNA of wild-type (WT) DT40 cells and each of the indicated cell lines, and hybridized sequentially with IGL and GAPDH probes. One representative example is shown. C) Average normalized steady-state transcript levels of IGL measured by Northern blot analyses obtained from at least four independent values per genotype. The value for wild-type (WT) DT40 cells is arbitrarily set to one. D) GCV was quantified by flow cytometry over four weeks starting from single cell clones. The average percentage of IgM+ cells from 12 subclones of each of the two independent 3kE lines is shown. E) Mutation event frequencies in the IGL (filled bars) and IGH (open bars) loci were determined by sequencing from two independent subclones after four weeks of continuous culture. The data for wild type (WT) and ΔME6K were obtained from [13]. Asterisks indicate p<0.005 in a Student's t-test.

To determine whether the 3′E enhancer is sufficient to support SHM/GCV in our DT40 system, we generated knock-in clones (3kE) in which the 3′E enhancer replaced the *cis*-regulatory elements of the *G. gallus IGL* locus. Again, the genotype of these 3kE lines were confirmed by Southern blot analyses (Fig. 3A). The steady state *IGL* transcript levels were again four-fold reduced compared to the ΔM line (Fig. 3B and 3C), comparable to what we observed in our ieMar knock-in lines (Fig. 2C). This corresponds to 77% of the *IGL* transcript levels in the wild-type cells, and hence, as discussed above, might be sufficient to support SHM/GCV. Furthermore, 3kE cultures showed no significant population of IgM^+^ cells, even over a growth period of four weeks (Fig. 3D). Sequencing analysis revealed a mutation frequency of 0.393×10^−4^ events/bp in the *IGL* gene of these cells (Fig. 3E), comparable to that of 0.363×10^−4^ events/bp seen in the ieMar genotype, and again below the background of this experimental system. Lastly, as expected, *IGH* diversification by SHM/GCV was still ongoing in the 3kE lines (Fig. 3E), suggesting that the absence of detectable AID-mediated sequence diversification in the *IGL* gene is simply due to a lack of a targeting element in our modified knock-in locus. This in turn infers, that when placed in the *IGL* locus of DT40 cells, the 3′E alone, just like the iE/MAR, works largely as a transcriptional enhancer with no detectable (based on the limit of our assays) targeting activity. This is again consistent with previous reports of 3′E-deficient mice, which were characterized by reduced levels of Igκ transcription and correspondingly mild SHM defects [20].

**Figure 4:**
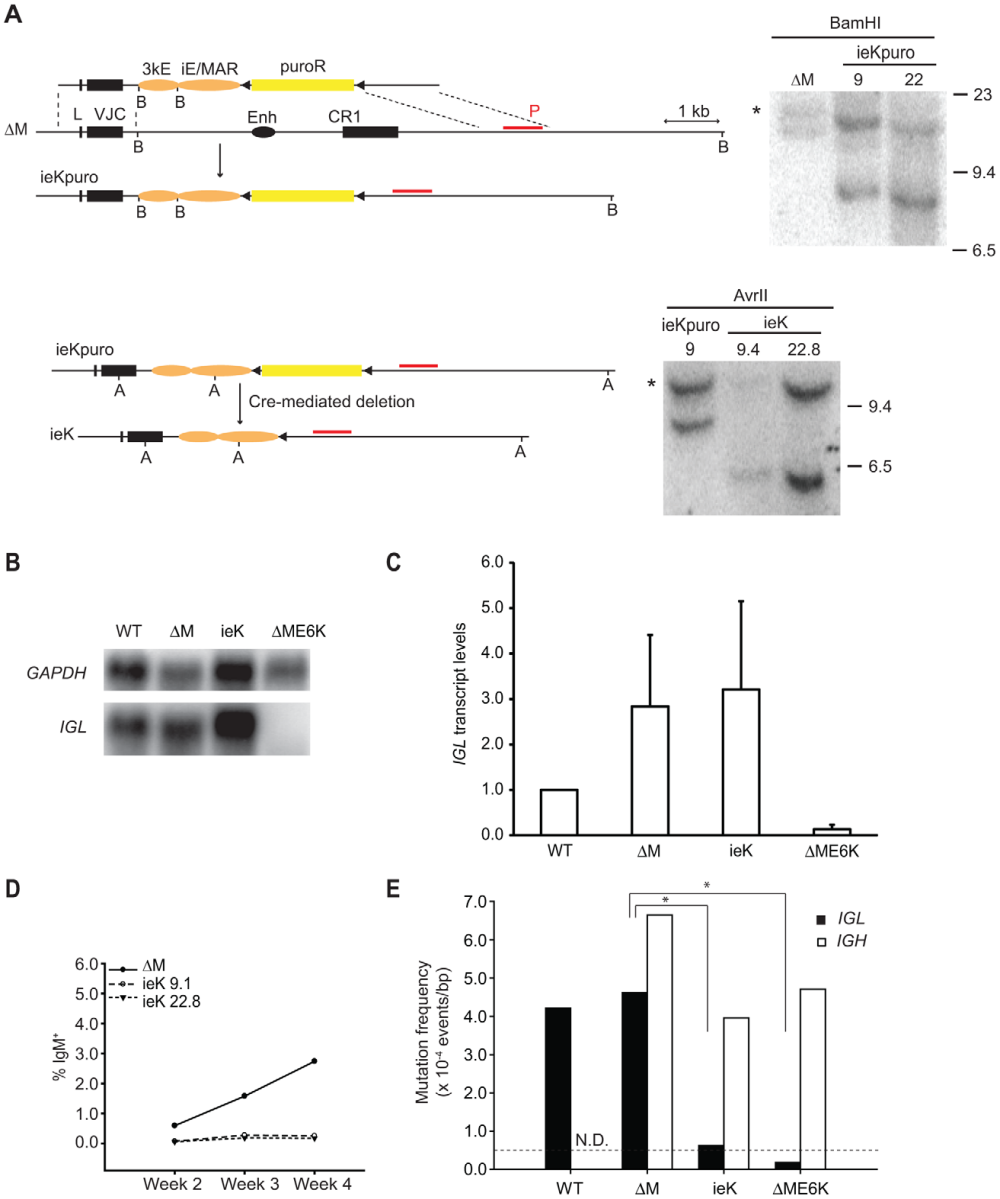
Analysis of double Igκ enhancer knock-in DT40 cells. A) Gene-targeting scheme with the murine iE/MAR and 3kE shown as orange ovals, for a detailed description of all elements see Fig. 1. The restriction sites are marked (A: AvrII, and B: BamHI), and the red line above the locus denotes the Southern blot probe (P). The asterisk in each Southern blot marks the band that originates from the non-rearranged allele. B) Northern Blots were run using 10 µg of total RNA of wild-type (WT) DT40 cells and each of the indicated cell lines, and hybridized sequentially with *IGL* and *GAPDH* probes. One representative example is shown. C) Normalized steady-state transcript levels of *IGL* were determined by Northern blots analysis. The level in wild-type cells is arbitrarily set to one, and an average of at least four data points obtained for each genotype is shown. D) Average percentages of IgM^+^ cells (twelve subclones per indicate clone) at each time point are shown for each genotype. E) Mutation event frequencies in the *IGL* (filled bars) and *IGH* (open bars) loci were determined by sequencing of the VJ or VDJ exon from two independent subclones of the ieK DT40 cells after four weeks of continuous culture. The data for wild type (WT) and ΔME6K were obtained from [13]. Asterisks indicate p<0.005 in a Student's t-test.

To address the formal possibility that both enhancers act in synergy to support AID-mediated diversification processes, double enhancer knock-in (ieK) clones were generated by gene-targeting, and verified by Southern blot analysis (Fig. 4A). Strikingly, simultaneous presence of both *M. musculus* Igκ enhancers supported *IGL* transcript levels comparable to those observed in the parental ΔM line (Fig. 4B and 4C). Culturing 24 sub-clones continuously for four weeks resulted in a minimal increase in the size of the IgM^+^ cell populations, indicating a dramatic reduction of GCV in the *IGL* locus of the ieK cells (Fig. 4D). To confirm this observation, we performed sequence analysis of the *IGL* gene, while the mutation frequency of 0.619×10^−4^ events/bp was higher than that observed in the individual enhancer knock-in lines (0.393×10^−4^ events/bp and 0.363×10^−4^ events/bp, respectively), it was still barely above the background of 0.5×10^−4^ events/bp (Fig. 4E). To confirm that these clones did not exhibit a general reduction of SHM/GCV due to reduced AID activity, the *IGH* variable region was sequenced as well. This analysis revealed clear evidence for levels of SHM/GCV comparable to those observed in the parental cell line in this gene locus (Fig. 4E). This suggests that even the presence of both *M. musculus* Igκ enhancers, the iE/MAR and the 3′E, is not sufficient to support high levels of AID-mediated sequence diversification in the context of the *G. gallus IGL* locus. As the *M. musculus* enhancer sequences used here were neither mutated nor truncated, they retained their native CAGGTG E-box binding sites. Thus the presence of these binding sites is not sufficient to efficiently target SHM/GCV at the robust levels observed in wild-type DT40 cells.

## Discussion

In summary, our results indicate that the iE/MAR and 3′E are active as transcriptional enhancers across species, as they are able to support transcription of the *IGL* locus in the *G. gallus* DT40 B cell line. Interestingly, however, they were unable to promote SHM. One obvious possibility is that the *M. musculus* elements do not work in the context of a *G. gallus* B cell nucleus. As both elements are able to function independently and cooperatively as transcriptional enhancers, we conclude that at least some of the transcription factor binding sites important for driving Ig gene transcription are utilized. Thus the species incompatibility of these *cis*-regulatory sequences would be largely confined to those binding site/DNA-binding factor pairs that are important for targeting AID activity. This would imply a provocative model in which the targeting of SHM to *Ig* loci involves species specific mechanisms. SHM is evolutionarily conserved and is critical for Ig gene diversification loci throughout the entire jawed vertebrate clade, and hence the existence of species specific targeting elements seems counterintuitive at first glance. Our data, however, are consistent with such model.

An alternative explanation for the lack of AID-mediated sequence diversification in the *IGL* gene of our knock-in lines is that the *M. musculus* enhancer elements are not sufficient or very inefficient in targeting SHM in general, which extends previous reports that neither of these enhancers by itself is strictly required for this process [20]. In this scenario, our observations imply that the key *cis*-regulatory sequences essential for SHM targeting reside in non-coding regions of the Igκ locus outside these well characterized transcriptional control elements. This model is fully compatible with the idea that both transcriptional enhancers, the iE/MAR and the 3′E, are involved in SHM, and that high levels of SHM activity require their functional interaction with the yet to be discovered targeting element. One candidate for such targeting element is the recently described Ed element, but its deletion had only modest effects on SHM [24].

Our results seem contradictory to a recent report by the Storb lab that three CAGGTG (E-box) sites within the *M. musculus* Igκ enhancers are sufficient to recruit SHM in DT40 cells [22]. The fundamental differences between these studies are: (1) the use of a sensitive GFP reporter-based transgenic system compared to our knock-in approach using the endogenous Ig gene context including the *G. gallus* Ig promoter, and (2) the sequencing of the GFP genes from selected GFP^+^ population (which may bias towards individual cells with higher levels of mutation) compared to the use of a bulk culture as source for genomic DNA. One plausible explanation for the discrepancies is that the transgenic system might emphasize the importance of the CAGGTG sites for SHM, but does not quantitatively address the magnitude of their role, as the double enhancer knock-in reported here showed only a minimal effect on SHM. This is consistent with the observation that E-box motifs are a common feature of genes that are transcribed in activated B cells and mutated at frequencies that are at least 50-fold lower than the cognate SHM target, the Ig genes, but still clearly higher than the remainder of the genome [9]. Alternatively, one could invoke the presence of putative negative regulatory elements residing in the *G. gallus IGL* locus that actively suppress the targeting activity of the *M. musculus* enhancers. While data in a recent study suggest that such elements exist (compare fragments M and N in Figure 3 of [14]), this DNA sequence is not present in any of our knock-in clones. In addition, non-coding sequences from Ig loci of other species are able to support normal levels of SHM/GCV in our system (N.R.K and S.D.F, unpublished data), and thus we consider the possibility of negative regulatory elements less likely. But at the current point in time we do not have any experimental data that would rule out this explanation.

In summary, our results are consistent with a model in which the traditional transcriptional Ig enhancers primarily function to support high levels of transcription of Ig genes, while the support of AID-mediated sequence diversification depends on additional mutation enhancer elements. The molecular mode of action of such *cis*-regulatory elements remains to be elucidated. A recent report suggests that the transcription elongation factor Spt5 acts an adaptor to recruit AID to actively transcribed gene loci and that AID is therefore more broadly distributed than previously anticipated [10]. This suggests that the targeting elements for SHM render AID highly active, and/or recruit error-prone DNA repair. Our model system represents a useful tool to reveal the identity of such *cis*-regulatory sequences within the large amount of as of yet uncharacterized non-coding DNA in Ig loci. But given the possibility of species specific targeting mechanisms our approach might not be applicable to elements from mammalian Ig loci. The identification of such *cis*-regulatory elements will in turn set the stage for subsequent studies to illuminate the underlying molecular mechanism of targeting.

## Materials and Methods

### Targeting constructs

The puromycin cassette of a modified pLoxPuro plasmid [25] was inserted as a *BamHI/BglII* fragment into ΔME6K vector [13] giving rise to the ΔME6K-BBHpuro plasmid. The iE/MAR and 3′E *M. musculus* enhancers were amplified by PCR from a plasmid (provided by Dr. F. Nina Papavasiliou, The Rockefeller University) and *M. musculus* genomic DNA using Phusion polymerase (New England Biolabs, Ipswich, MA) and primer pairs EiKr: 5′-acttggatccaactgctcactggatggtgg-3′/EiKf: 5′-acttggatccagcttaatgtatataatcttttag-3′ and 3kEr: 5′-gtacggatccagctcaaaccagcttaggcta-3′/3kEf: 5′-gtacggatccctagaacgtgtctgggccccatgaa-3′, respectively. The enhancers were subsequently inserted as BamHI fragments into the BamHI site of ΔME6K-BBHpuro to obtain the final targeting vectors. For the plasmid carrying both enhancers, the iE/MAR enhancer was first inserted as a *BamHI/BglII* fragment (amplified using primer pair EiKf/BglII-EiKr: 5′-acttagatctaactgctcactggatggtgg-3′) into ΔME6K-BBHpuro and the 3′E was subsequently inserted as a BamHI fragment.

### Tissue culture and gene-targeting

DT40 cells (provided by Dr. Jean-Marie Buerstedde, GSF, Munich, [26]) were grown in RPMI 1640 (Mediatech, Manassas, VA) supplemented with 10% fetal bovine serum (Invitrogen, Carlsbad, CA), 1% chicken serum (Invitrogen), 10 mM HEPES, 2 mM L-glutamine, and penicillin/streptomycin (Mediatech) at 41°C in 5% CO2. Transfections were performed in 4 mm cuvettes using 25–30 µg of linearized plasmid DNA in a Bio-Rad Gene Pulser (Bio-Rad, Hercules, CA) at 580 V, 25 µF, and ∞ Ω resistance. Stable transfectants were selected with 0.5 µg/ml puromycin (Invitrogen), and clones with targeted integrations were identified by Southern blot analyses. The Southern blot probes were amplified from genomic DNA by PCR using primer pair erar4: 5′-agcacagaacaggcacgtgct-3′/QG11R: 5′-gacgttgatgtgggacgatgtg-3′ (for probe P, see Figs. 2, 3, 4) or CCLF1: 5′-cccaccgtcaaaggaggagctg-3′/CHVLR1: 5′-cagtagatctttagcactcggacctcttcagg-3′ (for probe P2, see Figure 2), and body-labeled with [α-^32^P]dCTP using the NEBlot kit (New England Biolabs). Blots were hybridized overnight, and bands were visualized using a Phosphorimager (Molecular Dynamics). Cre-mediated deletion of puromycin resistance cassettes was done using a cell-permeable His-Tat-NLS-Cre fusion protein (where NLS is "nuclear localization signal") as described previously [13]), and confirmed by Southern blot analyses.

### Mutation analysis

Gene conversion and mutation analysis was performed as described previously [13]. Briefly, single cell clones were obtained by limiting dilution and cultured continuously for four weeks. Surface IgM was analysis by flow cytometry using a PE-conjugated anti-chicken IgM Ab (Southern Biotechnology, Birmingham, AL). VJ region sequences were amplified and sequence from the genomic DNA of two individual subclones (one per independent clone for each genotype) at the end of the four week period. Mutation frequencies were calculated as the ratio of the number of mutation events (both, templated as well as non-templated) to the total number of base pairs sequenced.

### Gene expression analysis

Total RNA was isolated using RNABee (Tel-Test, Friendswood TX) according to the manufacturer's protocol, and transcript levels were determined by Northern blot analyses. Probes for *IGL* and *GAPDH* were amplified by PCR using primer pairs CCLF1: 5′-cccaccgtcaaaggaggagctg-3′/CHVLR1: 5′-cagtagatctttagcactcggacctcttcagg-3′ and CHGAPDHF: 5′-accagggctgccgtcctctc-3′/CHGAPDHR: 5′-ttctccatggtggtgaagac-3′. Signals were detected using a Storm PhosphorImager (Amersham Biosciences, Piscataway NJ) and quantified using ImageQuant (Molecular Dynamics).
